# Hypocaloric Dieting Unsettles the Neuroenergetic Homeostasis in Humans

**DOI:** 10.3390/nu13103433

**Published:** 2021-09-28

**Authors:** Ewelina K. Wardzinski, Carolin Hyzy, Kai Uwe Duysen, Uwe H. Melchert, Kamila Jauch-Chara, Kerstin M. Oltmanns

**Affiliations:** Section of Psychoneurobiology, Center of Brain, Behavior and Metabolism, University of Luebeck, Ratzeburger Allee 160, 23538 Luebeck, Germany; CaroLange@gmx.de (C.H.); kai.duysen@uni-luebeck.de (K.U.D.); uwe.melchert@uni-luebeck.de (U.H.M.); kamila.jauch-chara@uksh.de (K.J.-C.); kerstin.oltmanns@uni-luebeck.de (K.M.O.)

**Keywords:** hypocaloric dieting, body weight, brain, humans, obesity, adenosine triphosphate, phosphocreatine, ^31^ phosphorus magnetic resonance spectroscopy

## Abstract

Background: The effects of low-calorie dieting in obesity are disappointing in the long run. The brain’s energy homeostasis plays a key role in the regulation of body weight. We hypothesized that the cerebral energy status underlies an adaptation process upon body weight loss due to hypocaloric dieting in humans. Objective: We instructed 26 healthy obese participants to reduce body weight via replacement of meals by a commercial diet product for two weeks. The cerebral energy status was assessed by ^31^ phosphorus magnetic resonance spectroscopy (31 PMRS) before and after low-caloric dieting as well as at follow-up. A standardized test buffet was quantified after body weight loss and at follow-up. Blood glucose metabolism and neurohormonal stress axis activity were monitored. Results: Weight loss induced a decline in blood concentrations of insulin (*p* = 0.002), C-peptide (*p* = 0.005), ACTH (*p* = 0.006), and norepinephrine (*p* = 0.012). ATP/Pi (*p* = 0.003) and PCr/Pi ratios (*p* = 0.012) were increased and NADH levels reduced (*p* = 0.041) after hypocaloric dieting. At follow-up, weight loss persisted (*p* < 0.001), while insulin, C-peptide, and ACTH increased (*p* < 0.005 for all) corresponding to baseline levels again. Despite repealed hormonal alterations, ratios of PCr/Pi remained higher (*p* = 0.039) and NADH levels lower (*p* = 0.007) 6 weeks after ending the diet. ATP/Pi ratios returned to baseline levels again (*p* = 0.168). Conclusion: Low-calorie dieting reduces neurohormonal stress axis activity and increases the neuroenergetic status in obesity. This effect was of a transient nature in terms of stress hormonal measures. In contrast, PCr/Pi ratios remained increased after dieting and at follow-up while NADH levels were still reduced, which indicates a persistently unsettled neuroenergetic homeostasis upon diet-induced rapid body weight loss.

## 1. Introduction

Obesity is a major public health problem, which has already reached an epidemic dimension [1,2]. The Organization for Economic Co-operation and Development stated in 2014 that almost 50% or more of the OECD population is overweight and the epidemic continues to grow [3]. Despite all efforts of obese individuals to lose body weight by various methods, the success is only of a transient nature. About 80% of people trying to reduce their body mass fail in terms of long-term weight loss maintenance [4]. Although the effect of subsequent body weight regain after hypocaloric dieting, even partly to a higher body mass than before the intervention (so-called yoyo effect), is well known, the underlying mechanisms remain unclear. Brain energy homeostasis is suggested to play a role in this context [5]. It has been repeatedly shown that obese individuals display reduced levels of high-energy phosphates, i.e., adenosine triphosphate (ATP) and phosphocreatine (PCr) [6,7,8], and moreover, demonstrate an impaired brain energy gain in response to increased blood glucose levels [8], both compared with normal weight controls. Moreover, the high-energy phosphate content predicts subsequent food consumption [9] and transcranial application of anodal direct currents reduces the overall caloric intake by 14% and diminishes self-reported appetite scores, possibly due to enhanced cerebral energy levels [10]. Although a reduction in calorie intake is recommended for body weight loss, it is not yet known how this affects brain metabolism.

Against this background, we hypothesized that two weeks of rapid body weight loss upon a hypocaloric diet would worsen the generally decreased cerebral energy status in obese individuals due to reduced brain energy supply across the blood–brain barrier in the form of circulating glucose. We further assumed that body weight regain due to a return to previous food intake habits after ending of the diet would restore brain energy supply and thereby neuroenergetic levels again. We therefore instructed 26 volunteers of both genders to replace meals by commercially available diet drinks for 14 days and monitored alterations in the cerebral energy status before and after the diet, as well as in a follow-up session 6 weeks thereafter. Because the nicotinamide adenine dinucleotide (NAD) pool is crucial for mitochondrial ATP synthesis [11] and the reduced form NADH predominates over NAD+ in mitochondria [12] playing a key role in cellular energy, we also monitored changes in NADH. Food intake behavior after hypocaloric diet and at follow-up was explored by standard test buffet [9].

## 2. Participants and Methods

### 2.1. Participants

Twenty-six healthy men (*n* = 12) and women (*n* = 14), aged 29.2 ± 1.01 years, body mass index 36.34 ± 0.96 kg/m^2^, participated in the study. The follow-up testing occurred 6 weeks after ending the diet. Participants were searched via newspaper, email, and flyer. A preliminary interview took place with each interested person, and all participants answered a questionnaire before measurements. Exclusion criteria were: BMI < 30 kg/m^2^, acute or chronic physical and mental illness, diabetes mellitus, exceptional physical or psychological stress, competitive sports, abuse of alcohol or addictive drugs, staying on a diet during the last 6 months before experimental testing, and regularly medication. We accepted sporadic pain medication, if it was taken at least 48 h before measurement and the anticonception pill in women. In women, the cycle day was additionally protocolized in order to detect possible hormonal bias. Participants were instructed to go to bed no later than 11 PM and not to perform intensive physical activity the day before the experimental testing. The study was performed in accordance with the Declaration of Helsinki (2013) of the World Medical Association and was approved by the ethics committee of the University of Lübeck. Each participant gave written informed consent.

### 2.2. Experimental Design

Each subject was tested before and after 14 days of diet-induced body weight loss. For follow-up testing, participants were invited 6 weeks after the end of the diet. On the days of experimental testing, participants reported to the Department of Neuroradiology in the morning after fasting for at least 8 h. Subsequently, a cannula was inserted into a peripheral vein on the back of the hand. Apart from key parameters of the glucose metabolism (plasma glucose, serum insulin, C-peptide), we determined circulating stress hormone (adrenocorticotropic hormone (ACTH), cortisol, epinephrine, norepinephrine) concentrations. On the first experimental day, commercial diet drinks (MEGAMAX^®^, MEGAMAX Holding B.V, The Netherlands) were handed out in two flavor variants (chocolate and vanilla) in order to ensure a hypocaloric energy balance during the 14 days of intervention. Both variants were comparable in terms of nutrient composition. According to the instruction sheet, drinks were prepared with low-fat milk. One portion of both diet drink contained 921 kJ. During the first week, participants were instructed to replace all main meals by diet drinks, while in the second week a usual lunch was allowed.

### 2.3. Standardized Buffet Testing

After 14 days of body weight loss and at follow-up examination, a standardized breakfast buffet was offered, from which participants were allowed to eat ad libitum during 30 min. To prevent overeating, participants were allowed to take home any remaining food. They were not aware of the hypothesized relationship between changes in overall cerebral energy content and food intake or of the food intake quantification by weighting buffet components before and after food intake. The following explanation was given to the participants: breakfast buffet was offered to compensate for the long fasting period as participants were expected to skip breakfast at home on the experimental days. Energy content (kJ) and macronutrient composition, i.e., the amount of carbohydrates, proteins, and fat, were evaluated using a professional nutrition software program (Prodi Version 5.9, Nutri-Science GmbH, Hausach, Germany). 

### 2.4. ^31^ Phosphor Magnetic Resonance Spectroscopy

^31^ P-MR spectroscopy of the motor cortex was conducted in a 3.0-Tesla magnetic resonance scanner (Achieva 3T, Philips Medical Systems, Best, The Netherlands) using a double-tuned ^1^H/ ^31^P-head coil (Advanced Imaging Research, Cleveland, Ohio). Before baseline ^31^P-MR spectra, scout images were taken. Thereafter, ^31^P-MRS was performed. A repetition time of 4500 ms with a three-dimensional chemical shift imaging sequence (6 × 5 × 3 voxel, 6 kHz bandwidth, 1024 data points, 4:44 min measuring time) was adjusted to obtain enough relaxation of the phosphorus metabolites. To gain a valid signal-to-noise ratio an elliptical k-space shutter was used, which skips the ky and kz pairs outside without loss of spatial resolution at the expense of CSI sharpness. In order to improve spectral resolution ^1^H-decoupling during excitation, nuclear Overhauser effect [13] and ^1^H-decoupling during receiving (wideband alternating-phase technique for zero-residual splitting) [14] was applied using the second channel of the head coil for transmitting on the ^1^H-resonance frequency. For our evaluation of the spectral data we employed a magnetic resonance user interface [15] with zero filling to 4096 data points, apodizing by a 45-Hz Lorentzian filter. Calculations of peak positions and intensities were defined using the Advanced Method for Accurate Robust and Efficient Spectral fitting (AMARES) algorithm [16]. ^31^P-MRS was used to measure the content of adenosine triphosphate (ATP) and phosphocreatine (PCr). ATP and PCr represent the overall cerebral high-energy phosphates. ATP levels were calculated from the sum of α-, β-, and γ-ATP [17,18]. In a bidirectional manner, ATP is formed by PCr and vice versa by creatine phosphokinase at a molar ratio of 1:1 [17]. Values represent the areas under the curves and therefore no units are indicated for ATP and PCr [19]. The ratios of ATP/inorganic phosphate (Pi) and PCr/Pi were evaluated because they are commonly used as indicators of the intracellular energy status and bioenergetics reserve in vivo [6,7,8,17,20]. In addition, we measured the cerebral content of the reduced form of nicotinamide adenine dinucleotide (NADH). NADH is one of the central electron donors in the oxidative phosphorylation in mitochondria providing electrons to the electron transport chain to generate most of the ATP. It represents a key sensor of cellular needs related to its glycolytic activity [21] and represents a signature of oxidative phosphorylation in the mitochondria of astrocytes and neurons [22]. 

### 2.5. Analyses of Blood Samples

Blood samples were centrifuged, and supernatants were stored at −85 °C until analysis. Plasma glucose concentrations were measured by the AU 5800 System (Beckman Coulter; inter-assay (CV) < 0.70%, intra-assay < 1.25%). Serum insulin, C-peptide, cortisol, and ACTH concentrations were determined by ElectroChemiLuminescence (ECL) immunoassay (Elecsys, cobas e 411 and 602, immunoassay-Systems, Roche Diagnostics, Germany; insulin: inter-assay coefficient of variation (CV) < 2.0%, intra-assay CV < 2.8%; C-peptide: inter-assay CV < 5.0%, intra-assay CV < 4.6%; cortisol: inter-assay CV < 2.8%, intra-assay CV < 1.7%; ACTH: inter-assay CV < 5.4%, intra-assay CV < 2.9%). Norepinephrine and epinephrine were measured by electrochemical detection (ECD) and an UV-detector for high-performance liquid chromatography (HPLC) by VWR Merck/Hitachi (norepinephrine inter-assay CV < 12.1%, intra-assay CV < 3.2%; epinephrine inter-assay CV < 9.9%, intra-assay CV < 1.8%).

### 2.6. Statistical Analyses

Statistical calculation was based on pairwise comparisons between single days (before and after 14 days of body weight loss, and at follow-up) using Student’s paired two-tailed *t*-test.

Furthermore, we performed the statistically appropriate Student’s paired one tailed *t*-tests to detect higher food intake in the follow-up testing because of the well-known weight regain after dietary weight loss as repeatedly demonstrated in clinical studies [23,24,25]. A < 0.05 significance level was chosen for all statistical tests. The baseline sample comprised *n* = 26, after 14 days of body weight loss *n* = 22, and at follow-up *n* = 19. After body weight loss by hypocaloric diet and at follow-up examination, 21 and 19 individuals participated in buffet testing, respectively.

## 3. Results

**Figure 1:**
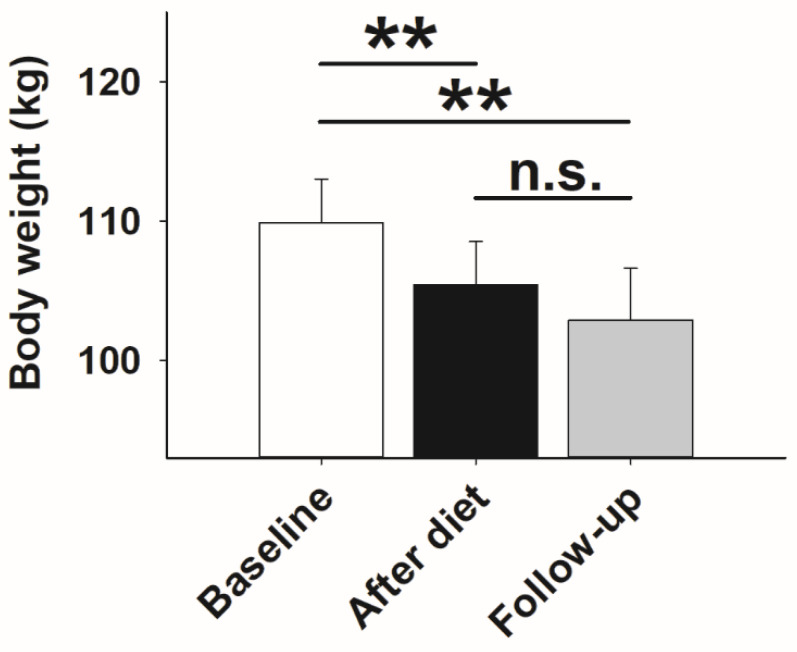
Body weight in kilograms (Mean values ± SEM) in obese participants at baseline (*n* = 26; white bar) after weight loss by hypocaloric diet (*n* = 26; black bar), and at follow-up (*n* = 19; grey bar). Solid line: Comparisons between single days were analyzed using the paired Student *t*-test. ** *p* < 0.010; not significant = n.s.

All participants of our study displayed a significant body weight loss after 14 days of dieting, which was evident after paired *t*-test comparisons before and after the intervention (*p* < 0.001; Figure 1). Weight loss remained evident at follow-up testing (*p* < 0.001; Figure 1), while body weight after dieting and at follow-up did not differ (*p* = 0.108; Figure 1). The average weight loss after 14 days of hypocaloric diet and at follow-up testing was 4.5 kg (±0.34 kg) and 3.6 kg (±0.76 kg), respectively.

### 3.1. Glucose Metabolism and Stress Axis Activity

**Figure 2:**
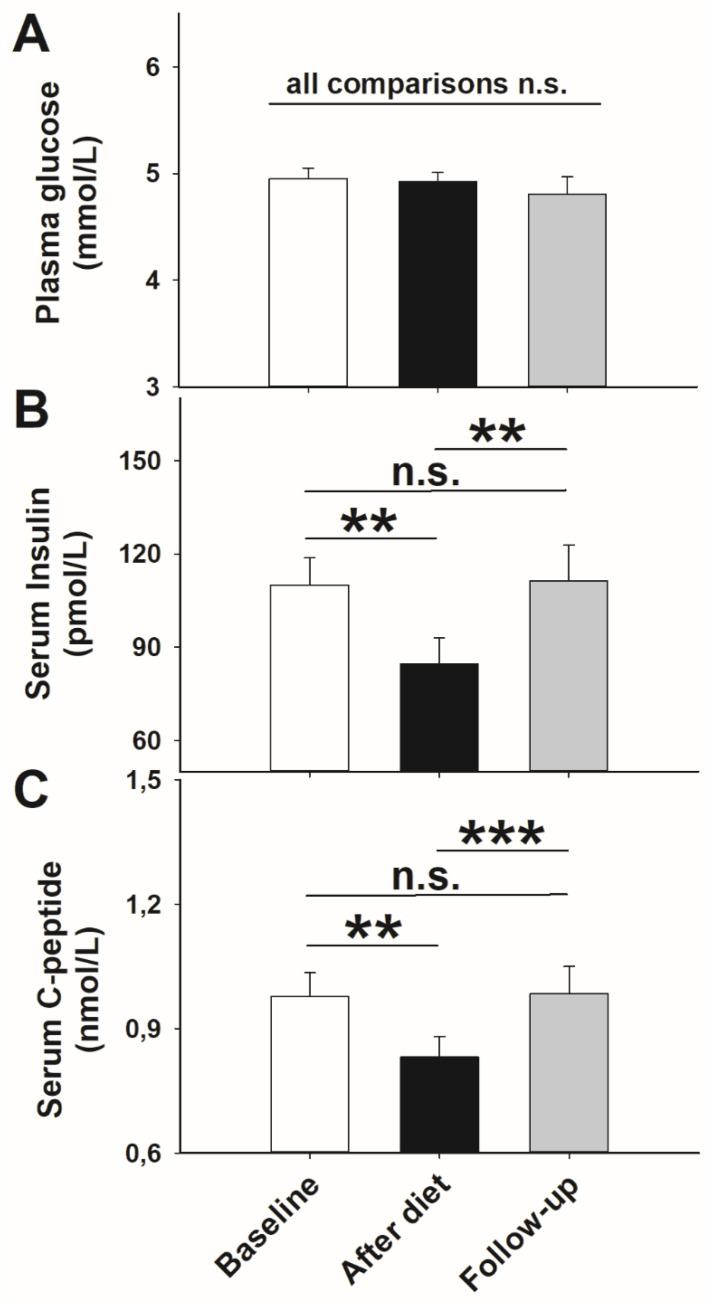
Mean values ± SEM of plasma blood glucose (**A**), serum insulin (**B**), and serum C-peptide (**C**) concentrations at baseline (*n* = 26; white bar) after weight loss by hypocaloric diet (*n* = 26; black bar), and at follow-up (*n* = 19; grey bar). Solid lines: Comparisons between single days were analyzed using a paired Student’s *t*-test. *** *p* < 0.005; ** *p* < 0.010; not significant = n.s.

Fasting plasma blood glucose concentrations were all within the physiological range of healthy individuals (4.90 ± 0.54 mmol/L) and did not differ between time points in all comparisons (*p* > 0.843 for all *t*-tests; Figure 2A). Serum insulin and C-peptide concentrations declined in the course of the study, i.e., they were both lower after 14 days of body weight loss compared with baseline (*p* = 0.002, *p* = 0.005, respectively, *t*-tests; Figure 2B,C). At follow-up, the levels of insulin as well as C-peptide were higher compared with the post-diet state (*p* = 0.005, *p* = 0.001, respectively, *t*-tests) and corresponded to baseline levels again (*p* = 0.983, *p* = 0.931, respectively, *t*-tests; Figure 2B,C).

**Figure 3:**
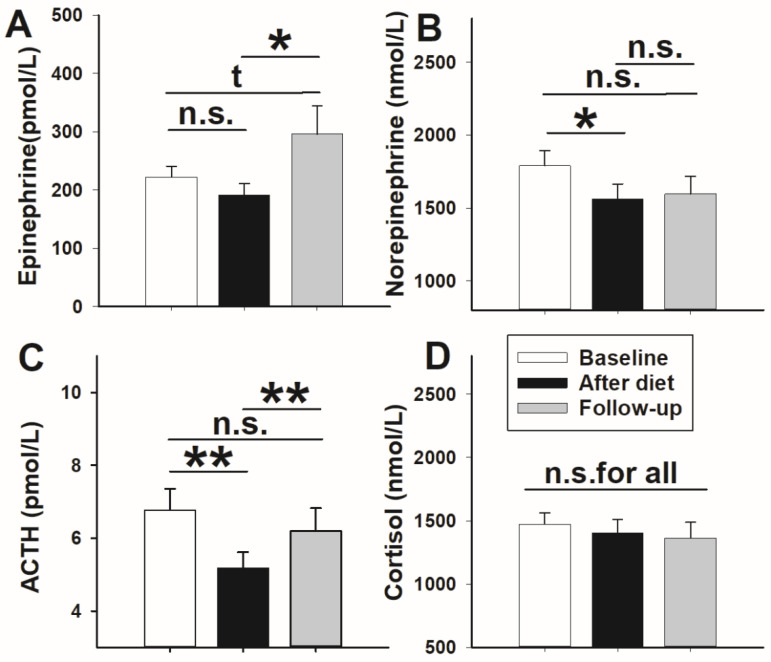
Mean values ± SEM of circulating epinephrine (**A**), norepinephrine (**B**), adrenocorticotropic hormone (ACTH) (**C**), and cortisol (**D**) concentrations at baseline (*n* = 26; white bar) after weight loss by hypocaloric diet (*n* = 26; black bar), and at follow-up (*n* = 19; grey bar). Solid lines: Comparisons between single days were analyzed using a paired Student’s *t*-test. * *p* < 0.050; ** *p* < 0.010; tp < 0.100; not significant = n.s.

In terms of stress axis activity, *t*-test analyses did not detect any effects of weight loss on epinephrine concentrations (*p* = 0.143, *t*-test; Figure 3A) but surprisingly demonstrated significantly increased values in follow-up measurements compared with the post-diet state (*p* = 0.036, *t*-test; Figure 3A). Comparisons between baseline and follow-up measurements revealed an increase by trend (*p* = 0.064, *t*-test; Figure 3A). In contrast, circulating norepinephrine concentrations significantly decreased upon weight loss compared with baseline (*p* = 0.012, *t*-test; Figure 3B). At follow-up, concentrations of norepinephrine did not differ to the post-diet state (*p* = 0.255, *t*-test; Figure 3B) or baseline (*p* = 0.491, *t*-test; Figure 3B), respectively.

Accordingly, analyses of serum ACTH concentrations showed lower values after 14 days of dieting compared with baseline measurements (*p* = 0.006, *t*-test; Figure 3C). At follow-up testing, concentrations of ACTH were higher than at the post-diet state (*p* = 0.005, *t*-test; Figure 3C) and reached baseline levels again (*p* = 0.745, *t*-test; Figure 3C). In contrast to the observed diet-induced effects in ACTH concentrations, cortisol levels did not differ in all testings (*p* > 0.464 for all, *t*-tests; Figure 3D).

### 3.2. High-Energy Phosphate Measurements

**Figure 4:**
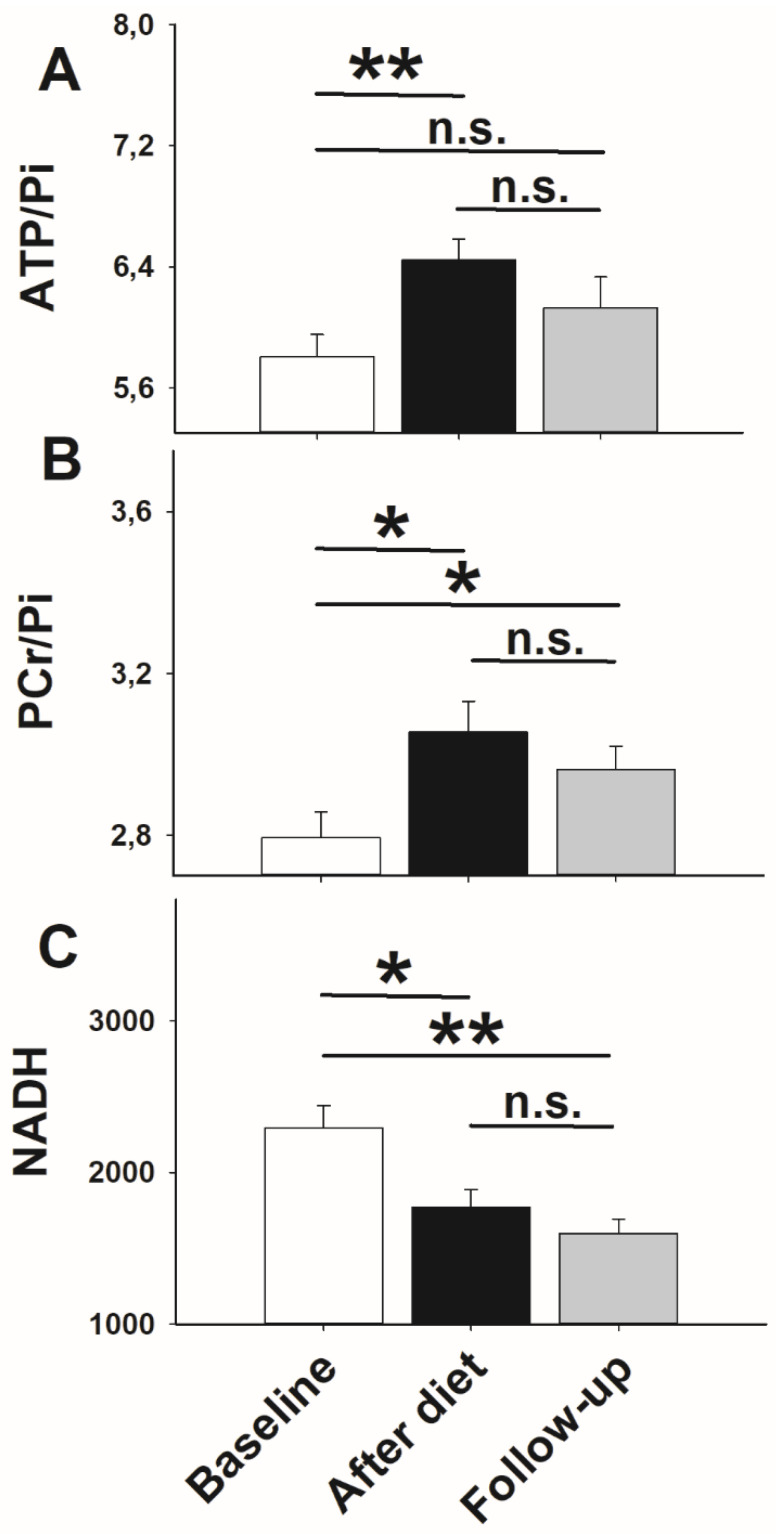
Impact of low-caloric dieting for 14 days on (**A**) adenosine triphosphate/inorganic phosphate ratios (ATP/Pi), (**B**) phosphocreatine/inorganic phosphate ratios (PCr/Pi), and (**C**) nicotinamide adenine dinucleotide phosphate content (NAD) in obese participants. Given are mean values ± SEM of ATP/Pi, PCr/Pi, and NAD before (*n* = 26) and after low-caloric dieting (*n* = 22) as well as at follow-up measurements (*n* = 19). Solid lines: Comparisons between single days were analyzed using a paired Student’s *t*-test. * *p* < 0.050; ** *p* < 0.010; not significant = n.s.

Statistical analyses of high-energy phosphates revealed that both ATP/Pi (Figure 4A) and PCr/Pi ratios (Figure 4B) were significantly higher after low-calorie dieting compared with baseline (*p* = 0.003, *p* = 0.012, respectively, *t*-tests). At follow-up, ratios of ATP/Pi (Figure 4A) and PCr/Pi ratios (Figure 4B) did not differ compared with the post-diet state (*p* = 0.099, *p* = 0.243, *t*-tests, respectively). However, ratios of ATP/Pi reached baseline levels at follow-up again (*p* = 0.168, *t*-test; Figure 4A), while PCr/Pi ratios remained higher (*p* = 0.039, *t*-test; Figure 4B). To ensure that the increase in high-energy phosphates is not based on reduced Pi, we also examined the stability of Pi during the entire experiment. Pi remained the same over time (*p* = 0.060, ANOVA RM).

NADH levels decreased after 14 days of low-calorie dieting (*p* = 0.041, *t*-test; Figure 4C) and remained lower at the follow-up compared with baseline (*p* = 0.007, *t*-test; Figure 4C). There was no difference between the post-diet state and follow-up measurements (*p* = 0.260, *t*-test; Figure 4C).

### 3.3. Standardized Buffet Tests

Buffet testing occurred after 14 days of dieting and at follow-up examination. The total amount of food intake after 14 days of dieting (2812 ± 232 kJ) did not statistically differ compared with the follow-up testing (3092 ± 248 kJ; *p* = 0.135, *t*-test). Analyses of respective macronutrient composition revealed a higher carbohydrate intake by strong trend in the follow-up testing (1473 ± 120 kJ) compared with testing after 14 days of low-calorie dieting (1275 ± 100 kJ; *p* = 0.054, *t*-test). Fat and protein consumption was similar directly after body weight loss (1128 ± 107 kJ, 409 ± 38 kJ) compared with follow-up testing (1224 ± 129 kJ, 392 ± 37 kJ; *p* = 0.249, *p* = 0.460, *t*-test, respectively). Due to a relatively high drop-out rate in the follow-up buffet test, the power was probably too small to detect statistical significance (Typ-II error).

## 4. Discussion

Our data confirm previous insight that rapid body weight loss by a two-weeks hypocaloric diet affects a number of hormonal parameters involved in glucose metabolism and stress perception. Despite stable blood glucose concentrations, dieting decreased circulating insulin and C-peptide levels, which is in line with previous observations [26,27,28]. These findings have been explained by improved insulin sensitivity due to hypocaloric dieting [29,30]. Moreover, our stress hormonal measurements revealed a reduced activity in terms of norepinephrine and ACTH upon diet-induced weight loss. This finding is in accordance with a previously revealed association between a declined urinary norepinephrine excretion and the maintenance of a 10% body weight reduction [31], as well as the observation of a relationship between reduced circulating serum insulin and decreased arterial norepinephrine concentrations in humans upon weight loss [32]. 

However, the main finding of our study is the considerable influence of hypocaloric dieting on the brain‘s energy homeostasis. In contrast to our hypothesis, we surprisingly found a significant increase in cerebral high-energy phosphate levels in response to two weeks of caloric restriction. In the attempt to explain this finding, at first sight one may speculate that the hypocaloric diet could have induced a “ketolytic” metabolism as known from low carbohydrate/high fat dieting [33,34,35], and thereby ketone bodies as alternative energy source for the brain have simply boosted the neurenergetic levels in our study. However, the macronutrient composition of our diet consisting of high carbohydrate, moderate protein, and low-fat nutrients contradicts this assumption. It rather appears conceivable that hypocaloric dieting of participants with an energetically undersupplied brain, as known from obesity [6,7,8], activates some kind of neuroprotective mechanism to prevent any further neuroenergetic drop under conditions of reduced energy supply through constricted nutrition. This is in line with previous findings of an increase in brain energy content upon a short period of hypoglycemia in humans [36], and enhanced brain ATP production upon caloric restriction in mice [37]. The brain is apparently able to activate specific mechanisms in order to ensure its own energy demand not only during short periods of hypoglycemia but actually during long-lasting intervals of reduced caloric intake. In this context, glucose transporter (GLUT) regulation is crucial, which mediates glucose uptake of organs and tissues by facilitated diffusion. In terms of the brain, GLUT 1 and GLUT 3 enable more than 95% of the glucose transfer from the peripheral blood circulation [38]. Previous findings demonstrated an increase in GLUT 1 expression in the brains of caloric restricted mice [39]. On the other hand, chronically elevated blood glucose levels, which occur in obesity and diabetes mellitus, lead to a downregulation and a decrease in the capacity to transport glucose across the blood–brain barrier via glucose transporters. Moreover, this leads to an increase in cerebral glucose utilization in a neuroprotective way [40,41,42]. These neuroprotective mechanisms serve to avoid too-high glucose concentrations within the brain because already short hyperglycemia leads to oxidative damage and apoptosis in neurons [43] and causes global cerebral atrophy, or mitochondrial dysfunction [44]. Calorie-reduced dieting and related lowered blood glucose concentrations may therefore increase the expression and/or the activity of glucose transporters and optimize the cerebral energy supply within narrow confines. This would explain our observation of increased cerebral energy levels after 14 days on a low-caloric diet. On the other hand, previous work also showed that an increased high-energy phosphate content was associated with decreased food intake [9]. 

However, in this context one must focus on another result of our study: we found that NADH levels remained lowered after ending of the diet, while ATP/Pi ratios returned to baseline levels again. This opposing effect may indicate a persistently unsettled brain energy homeostasis after hypocaloric dieting. The mitochondrial NAD pool (including NAD+ and NADH) is critically important for the biochemical synthesis of ATP, and is sufficiently robust to preserve oxidative phosphorylation [11]. NADH is a crucial coenzyme in the ATP synthesis. It exists in two forms within the cell: NAD+ and NADH, and reflects glycolysis and oxidative phosphorylation activity in the mitochondria of astrocytes and neurons [22]. Therefore, NAD+ is a vital cofactor that can link the energy status with adaptive cellular and organismal responses [45]. The observed lowered cerebral NADH levels and simultaneously baseline returned ATP/Pi ratios at follow-up thereby indicate a reduced cerebral energy synthesis capacity upon low-caloric dieting.

Overall, our neuroenergetic data, i.e., reduced cerebral mitochondrial energy synthesis capacity, suggest persisting detrimental effects of dieting on the brain homeostasis, which must consequently be compensated through an increased energy supply in order to restore baseline brain energy levels and thereby a balanced energy homeostasis again. This assumption is supported by the finding of a strong trend towards increased carbohydrate craving in the follow-up testing. In fact, Kistenmacher et al., recently demonstrated a relationship between lowered cerebral NADH levels and increased food ingestion [46]. A repetitive or persistent NADH decrease induced by repetitive low-caloric dieting could therefore lead to boosted food craving and thereby consumption to satisfy the cerebral energy needs. This mechanism may provide an explanation for the frequently observed so-called yo-yo effect. This effect is known as weight cycling and describes repeated periods of initially successful weight loss followed by regain even beyond the initial body weight [47].

Our findings of reduced cerebral NADH levels 6 weeks after hypocaloric diet and the fact that lowered brain ATP levels are well-known in obesity [6,7,8] may reflect an impaired oxidative phosphorylation, i.e., energy synthesis within the mitochondria in neurons. This is in line with several studies demonstrating a link between obesity and mitochondrial dysfunction [48,49]. Canto and colleagues described a close relationship between the NAD+ bioavailability and obesity [50]. Their work showed that the natural NAD+ precursor is a powerful nutritional supplement to protect against high-fat diet-induced obesity, which is characterized by defective mitochondrial functions [50]. Although our study does not allow for any conclusions about pathological processes at the molecular level, our results indicate a prolonged impairment in cerebral energy homeostasis after 14 days of low-caloric dieting. 

However, this reasoning remains speculation and requires some further research at this point. One limitation of our study is certainly the high drop-out rate of participants in the standardized buffet testing at follow-up (but not in the brain measurements), which leads to unbalanced samples sizes and limits the power of some statistical analyses. Given that a significant increase in food intake after low-caloric dieting occurs, we could have missed this effect due to an insufficient sample size. In this context, the observed trend towards an increased carbohydrate intake in the follow-up may be interpreted as a clear indication of a diet-induced alteration in food intake behavior due to a modified brain metabolism. Furthermore, the noninvasive semiquantitative ^31^P-MR spectroscopy method allows reliable statements about the relative, but not the absolute content, of high-energy phosphates and their changes over time in vivo. Thus, changes in high-energy ratios are not mandatory linearly correlated to changes in high-energy content. Furthermore, it is difficult to conclude from an increase in ATP or PCr content whether this is due to enhanced synthesis or reduced oxidation of ATP and PCr. However, building ratios with Pi in this context implies that enhanced synthesis may underlie this effect, because Pi is in a direct chemical relationship with ATP (ATP ↔ ADP + Pi +31 kJ/mol), ATP hydrolysis leads to an increase in Pi content and vice versa. An increase in high-energy phosphate to Pi ratios therefore indicates a shift of the chemical equation towards ATP, i.e., increased energy synthesis [51]. In line, our observed increase in high energy ratios is not due to reduced Pi values and therefore we can assume an increased energy synthesis.

Moreover, it must be mentioned that some aspects of our study may also limit the interpretation of our NADH data. One may question the reliability of these data because of a too low magnetic field strength (3T) in our scanner. However, there is evidence from current international literature, which demonstrates that human brain measurements with magnetic field strength ranging from 3-7T are all comparably reliable for conclusions in this context [52]. 

An additional point of criticism may be that the coil would sense different loading depending on the subjects and their position inside the coil. This in turn may give different signal intensity even for the same subject at different time points. In fact, because our participants were in the scanner for almost 30 min, we cannot completely exclude that potential movements may have influenced the spectral data. On the other hand, our participants’ heads were fixed in a head shell, which allowed only very limited movements. Additionally, insufficient accuracy of NADH measurements would not have led to statistically significant differentiation of the signals, which we could show in our results. Another important consideration for further studies could be the additional measurement of NAD+ as well as the formation of ratios from NAD/ NADH to gain more in-depth insights into cerebral energy metabolism.

## 5. Conclusions

In summary, our data provide evidence that low-caloric dieting for two weeks transiently increased cerebral high-energy phosphate levels in obese humans. In the long term, our data indicate that a hypocaloric diet may have detrimental consequences on the brain’s energy homeostasis in terms of a decreased neuronal energy synthesis capacity. In conjunction with the insight that caloric restriction is not successful in order to lose weight in the long run, calorie-reduced diets should be scrutinized as a therapy option in obesity.

## Data Availability

Data is contained within the article and could be presented on request.
